# Attribute based cross classification analyses from the BRIGHTEN study reveal that therapeutic responsiveness to erythropoiesis stimulating agents predicts cardiorenal prognosis in renal anemia

**DOI:** 10.1038/s41598-025-05762-y

**Published:** 2025-07-02

**Authors:** Hiroshi Kataoka, Terumasa Hayashi, Masaomi Nangaku, Ichiei Narita, Tatsuo Kagimura, Kosaku Nitta, Junichi Hoshino

**Affiliations:** 1 Department of Nephrology, Tokyo Women’s Medical University, 8-1 Kawada-cho, Shinjuku-ku, Tokyo, 162-8666 Japan; 2 Department of Clinical Engineering, Faculty of Human Care at Makuhari, Tohto University, Chiba, Japan; 3 Osaka General Medical Centre, Osaka, Japan; 4 Graduate School of Medicine, The University of Tokyo, Tokyo, Japan; 5 Graduate School of Medical and Dental Sciences, Niigata University, Niigata, Japan; 6 The Translational Research Center for Medical Innovation, Hyogo, Japan

## Abstract

Renal anemia outcomes are influenced by sex and age; however, current guidelines for renal anemia lack sex- or age-specific standards for initiating treatment. Recently, the concept of attribute-based medicine has gained attention, with an emphasis on personalized approaches based on patient characteristics. As prognostic factors for renal and cardiovascular outcomes may vary between men and women, as well as between younger and older patients with chronic kidney disease (CKD), we aimed to investigate anemia treatment-related indicators influencing kidney disease progression and cardiovascular events across four age and sex attributes (cross-classified by sex and age < or ≥ 65 years). A total of 1,671 patients with CKD and renal anemia from the BRIGHTEN study, who were initiated on an erythropoiesis-stimulating agent (ESA) according to Japanese guidelines for renal anemia recommending initiating treatment when the hemoglobin (Hb) level is 11 g/dL), were analyzed using the ESA resistance index-1B (ERI-1B) and the initial ESA response index (iEResI). Baseline Hb levels were similar across all sub-groups (9.7–9.9 g/dL), and kidney function was lowest in younger men (estimated glomerular filtration rate, 15.6 ml/min/1.73 m^2^) and highest in older women (estimated glomerular filtration rate, 19.4 ml/min/1.73 m^2^). Darbepoetin alfa doses during 12 weeks were lowest in younger men (0.79 μg/kg) and highest in older women (1.08 μg/kg). Renal outcomes were the poorest in younger men and the best in older women. Cardiovascular outcomes were the poorest in older men and the best in younger women. Multivariable Cox analyses showed that baseline hemoglobin levels (hazard ratio [HR], 0.77; *P* < 0.001) and the iEResI (HR, 0.76; *P* < 0.001) were associated with kidney prognosis across the cohort, whereas ERI-1B was not. When stratified by cross-classification, kidney prognosis correlated with iEResI (HR, 0.63; *P* = 0.019) in younger men and with ERI-1B (HR, 1.10; *P* < 0.001) in older women. Renal outcomes were linked to hemoglobin levels and ESA responsiveness in younger men with the poorest outcomes and to ESA resistance in older women with the best outcomes. This study revealed that renal outcomes correlated with Hb levels and ESA responsiveness in patients with CKD and anemia, especially in younger men with poor renal outcomes and with ESA resistance in older women with the best renal outcomes. Cross-classification helps identify specific patient attributes that should be targeted for optimizing anemia treatment in CKD.

## Introduction

Personalized medicine, also known as precision medicine, has been developed in parallel with patient-centered care^1–4^, emphasizing treatments tailored to individual characteristics^5,6^. Recent advancements in artificial intelligence and machine learning have further supported this approach^7–9^. However, challenges remain^7,10^, particularly for patients with chronic kidney disease (CKD), who often present with multiple risk factors due to the chronic nature of their condition^11,12^. Subgroup analyses can provide valuable insights for developing effective, personalized, evidence-based care^1,2,13^, highlighting the benefits of examining differences among sub-cohorts^6,14–16^. For instance, in the treatment of hyperuricemia in patients with CKD, therapeutic efficacy was found to depend on patient attributes, with febuxostat shown to mitigate the decline in kidney function in patients without proteinuria^17^. These findings suggest that attribute-based medicine (ABM)^12,17^ could represent an essential first step toward developing personalized regimens for patients with CKD^10^. ABM is a medical approach that emphasizes tailoring care by focusing on patient-specific attributes or disease characteristics to provide precise and personalized treatment^10,12,17^. Subgroup analyses informed by ABM are emerging as a crucial step toward developing personalized regimens for patients with chronic diseases such as CKD^10,12,17^.

Sex is a particularly important attribute in kidney disease^18–20^, with sex-based differences in anemia severity among patients with CKD having been reported^21–25^. The 2020 “Kidney Disease: Improving Global Outcomes” conference emphasized the need for individualized anemia management in patients with CKD, highlighting the importance of tailoring treatments based on medical conditions, age, sex, and comorbidities^26^. However, existing anemia guidelines have not established sex-specific treatment standards, reflecting a lack of evidence on anemia management for both men and women. To address this gap, it is crucial to build a robust body of evidence on anemia in both sexes to guide the development of future sex-specific treatment standards. Consequently, sex-specific approaches to anemia management are becoming increasingly vital for effectively addressing renal anemia.

**Fig. 1:**
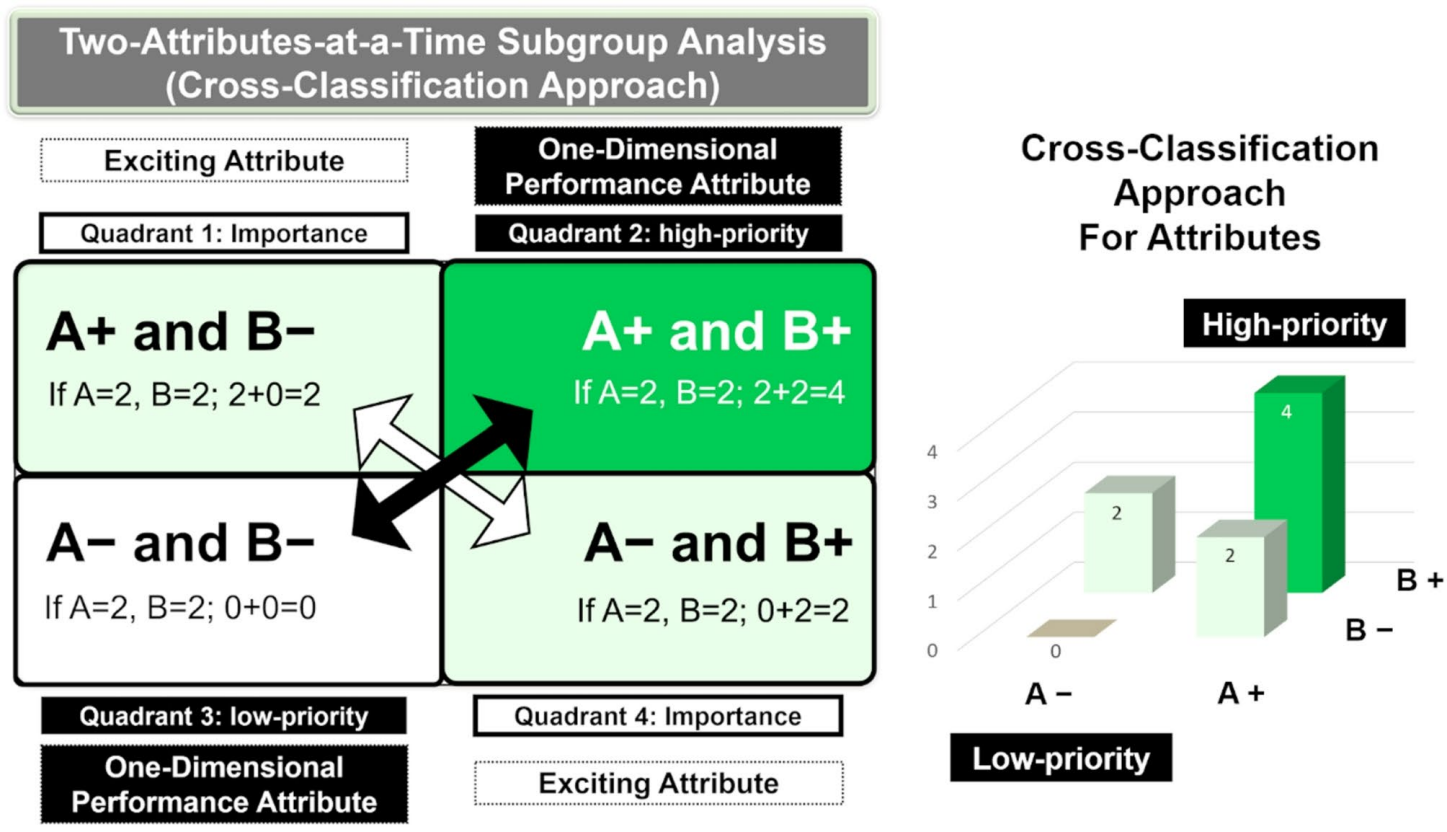
Attribute-based cross-classification approach (Importance Grid Analysis). Attributes positioned on the diagonal represent the most opposite characteristics. If attributes A and B are deemed important, an attribute containing both A and B will be considered the most important, while an attribute lacking both A and B will be regarded as the least important. For instance, if values are assigned to attributes A and B, with both set to 2, the bottom left section will have a value of 0 and the upper right section will have a value of 4.

Two-attribute-at-a-time subgroup analysis (cross-classification) is frequently used in marketing research and other fields, offering benefits for ABM^12,17,27^. Patients often possess multiple overlapping attributes, which limits the utility of traditional subgroup analyses that classify individuals based on a single variable (e.g., male vs. female or older adults vs. young)^2,12^. Such one-variable-at-a-time comparisons typically result in subgroups that closely resemble the overall cohort because they fail to simultaneously account for the complex interplay of multiple characteristics^12,28^. Certain pathological conditions can only be identified by cross-classifying individuals into four groups. In cross-classified models, the main diagonal elements show direct effects, while off-diagonal elements represent indirect effects, both of which are crucial for distinguishing sub-cohorts^29^. As shown in Fig. 1, this structure identifies interactions and reveals high or low-priority attributes^17,27^. Regarding kidney prognosis, notable physiological and pathological distinctions between young males and older females imply the need for tailored risk management and treatment approaches for each group^12,30^. Renal anemia is also affected by age and sex^31^. Therefore, we hypothesized that the efficacy of treatment for anemia is influenced by sex and age, with treatment being more beneficial for male patients with CKD under 65 years of age^32^.

The multicenter, prospective BRIGHTEN study aimed to establish indices of darbepoetin alfa (DA) hyporesponsiveness associated with poor renal and cardiovascular outcomes in non-dialysis patients with CKD^33^, introducing erythropoiesis-stimulating agent (ESA) resistance and ESA reactivity indices to detect individual hematopoietic responses^34^. The BRIGHTEN study presents renal outcomes and cardiovascular disease (CVD) events in patients with CKD in a real-world clinical setting in Japan. In the present study, we conducted attribute-based research within the BRIGHTEN study framework to assess how ESA resistance and reactivity indices influence CKD progression and cardiovascular events in cohorts cross-classified by sex and age.

## Methods

### Study design

**Fig. 2:**
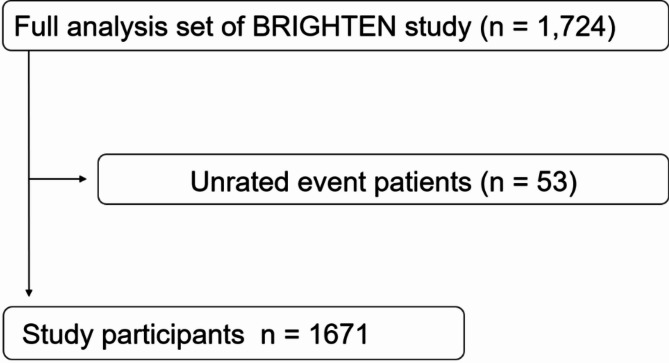
Flow chart of patient selection.

The detailed BRIGHTEN study design, criteria, and ethics are extensively outlined in previous reports^33,34^. Conducted within Japan’s healthcare framework, the BRIGHTEN study adhered to the Declaration of Helsinki and Japan’s Ethical Guidelines on Clinical Studies. The participants provided written informed consent, and the protocol was approved by the review boards of Nagoya University (Approval Number: 2014-0027) and its affiliated facilities (Niigata University, 2023–0014; Tokyo Women’s Medical University, C23-043). This study was registered with ClinicalTrials.gov (NCT02136563; registration date: May 8, 2014) and UMIN-CTR (UMIN000013464; registration date: May 2, 2014). Patients aged ≥ 20 years with an estimated glomerular filtration rate (eGFR) of < 60 mL/min/1.73 m^2^ (calculated using a previously reported Japanese equation ^35^) who presented with renal anemia (hemoglobin [Hb] < 11 g/dL) were enrolled from June 2014 to September 2016 and observed for 96 weeks after DA administration^33^. DA was initially administered at 30 μg every 2 weeks, with dose adjustments made to maintain Hb ≥ 11 g/dL at the discretion of the facility physicians^33^. A total of 1,671 patients were enrolled after excluding 53 patients whose events could not be evaluated in the preceding study^36^ (Fig. 2). The patients were cross-classified by age and sex as follows: 202 women aged < 65 years, 481 women aged ≥ 65 years, 246 men aged < 65 years, and 742 men aged ≥ 65 years.

### Outcome evaluation

The primary endpoint comprised renal events indicating deterioration in renal function, specifically initiation of maintenance dialysis, kidney transplantation, a 50% decrease in eGFR, or an eGFR of ≤ 6 mL/min/1.73 m^2,33,36^. The secondary endpoint consisted of fatal or non-fatal CVD events, as previously defined in the BRIGHTEN study^33,36^.

Based on existing literature^12,36^, the following variables were explored as potential prognostic factors for each event: age, sex, diabetes mellitus, hypertension, dyslipidemia, smoking, baseline Hb level, eGFR, urinary protein-to-creatinine ratio, ESA resistance index-1B (ERI-1B)^34^, and initial ESA response index (iEResI)^34^. The formulas for ERI-1B and iEResI are as follows^34^:

$${\text{ERI}} - {\text{1B}} = {\text{Dose }}\;{\text{of}}\;{\text{ DA}}\;{\text{ at}}\;{ 12}\;{\text{ weeks}}\;\left( {\mu {\text{g}}} \right){\text{/Concentration}}\;{\text{ of}}\;{\text{Hb}}\;{\text{at}}\;{12}\;{\text{weeks}}\;\left( {\text{g/dL}} \right),$$

$${\text{iEResI}} = \Delta {\text{Hb}}_{{0 - {12}}} \left( {\text{g/dL}} \right) \times {\text{body}}\;{\text{weight}}\;\left( {{\text{kg}}} \right){\text{/Total}}\;{\text{dose}}\;{\text{of}}\;{\text{DA}}\;{\text{during}}\;{12}\;{\text{weeks}}\;\left( {\mu {\text{g}}} \right).$$

### Statistical analyses

Baseline characteristics are expressed as mean ± standard deviation, median (interquartile range), or number (percentage). Differences between the groups were compared using analysis of variance for continuous variables, Kruskal–Wallis test for variables with skewed distributions, as indicated by the median [interquartile range] values, and χ^2^ test for frequency distributions. Cox proportional hazards models with covariates were used to explore the prognostic factors influencing the incidence of renal function deterioration and CVD events. Interactions between sex, age category, and each variable of interest were considered by adding each category and the corresponding interaction term to the multivariable analyses. Standard methods were employed to estimate the sample size for multivariable logistic regression analyses, requiring five or more outcomes per independent variable^37,38^. Cumulative survival curves were estimated using the Kaplan–Meier method, and yearly incidence rates with 95% confidence intervals (CIs) were calculated. Given the exploratory design of this study, we did not adjust for the multiple variables. Analyses were conducted using SAS v.9.4 (SAS Institute, Inc., Cary, NC, USA), with statistical significance defined as *P* < 0.05 (two-tailed).

## Results

### Patient characteristics

Patient characteristics are presented in Table 1. The baseline Hb level was 9.8 g/dL and was almost similar in all groups (range: 9.7–9.9 g/dL, lowest in women aged ≥ 65 years). The median eGFR was 18.2 mL/min/1.73 m^2^ at baseline and varied between the groups (*P* < 0.001): women aged < 65 years (17.5 mL/min/1.73 m^2^), women aged ≥ 65 years (19.4 mL/min/1.73 m^2^), men aged < 65 years (15.6 mL/min/1.73 m^2^), and men aged ≥ 65 years (18.7 mL/min/1.73 m^2^). The average DA dose was 0.93 μg/kg for 12 weeks and varied between the groups (*P* < 0.001): women aged < 65 years (0.97 μg/kg), women aged ≥ 65 years (1.08 μg/kg), men aged < 65 years (0.79 μg/kg), and men aged ≥ 65 years (0.79 μg/kg). The DA dose was lowest in men aged < 65 years and highest in women aged ≥ 65 years. After 12 weeks of DA treatment, the Hb level at week 12 was 11.0 g/dL, an increase of 1.2 g/dL from baseline. Levels were comparable among the groups (range: 10.9–11.1 g/dL, lowest in women aged ≥ 65 years; Δ: 1.2–1.3 g/dL). Regarding the DA response and DA resistance, the median iEResI was highest in men aged < 65 years (0.58) and lowest in women aged ≥ 65 years (0.43) (*P* < 0.001), suggesting that the DA response was highest in younger men and lowest in older women. In contrast, no significant between-group differences were observed for ERI-1B.

**Table 1:** Patient characteristics (entire cohort, and sub-cohorts based on sex and age based cross-classification).

Variable	Entire cohort n = 1671	Women, < 65 years n = 202	Women, ≥ 65 years n = 481	Men, < 65 years n = 246	Men, ≥ 65 years n = 742	*P*–value
***ESA-related factors***						
Total dose of DA during 12 weeks/body weight (μg/kg)	0.93 (0.63)	0.97 (0.60)	1.08 (0.73)	0.79 (0.49)	0.87 (0.60)	< 0.001
iEResI	0.51 [0.25, 0.82]	0.49 [0.28, 0.69]	0.43 [0.17, 0.71]	0.58 [0.26, 0.99]	0.55 [0.29, 0.89]	< 0.001
ERI-1B	5.17 (3.71)	5.35 (3.72)	5.33 (3.97)	5.12 (3.57)	5.03 (3.56)	0.520
***Clinical findings***						
Age (years)	69.94 (11.97)	53.95 (8.43)	76.24 (6.93)	54.16 (8.54)	75.44 (6.34)	< 0.001
Sex (male), n (%)	988 (59.1)	0 (0.0)	0 (0.0)	246 (100.0)	742 (100.0)	< 0.001
Body weight, (kg)	58.73 (12.57)	56.12 (13.19)	50.65 (10.16)	69.20 (14.57)	61.21 (9.41)	< 0.001
Body mass index, (kg/m^2^)	23.12 (4.04)	22.99 (5.09)	22.68 (4.19)	24.36 (4.63)	23.04 (3.28)	< 0.001
Systolic arterial pressure (mmHg)	134.19 (19.02)	131.62 (21.10)	135.43 (18.02)	134.55 (19.51)	133.96 (18.87)	0.139
Diastolic arterial pressure (mmHg)	71.32 (12.28)	75.67 (12.91)	70.15 (11.92)	76.72 (12.10)	69.19 (11.60)	< 0.001
Smoking status (current or ever), n (%)	795 (49.6)	47 (24.5)	61 (13.4)	166 (70.3)	521 (72.3)	< 0.001
Etiology of CKD						< 0.001
Diabetic nephropathy, n (%)	459 (27.6)	45 (22.4)	88 (18.4)	116 (47.3)	210 (28.5)	
Chronic glomerulonephritis, n (%)	391 (23.5)	56 (27.9)	114 (23.8)	43 (17.6)	178 (24.1)	
Nephrosclerosis, n (%)	392 (23.6)	17 (8.5)	145 (30.3)	26 (10.6)	204 (27.6)	
Polycystic kidney disease, n (%)	95 (5.7)	40 (19.9)	23 (4.8)	13 (5.3)	19 (2.6)	
Other, n (%)	325 (19.6)	43 (21.4)	108 (22.6)	47 (19.2)	127 (17.2)	
***Comorbidities***						
Diabetes, n (%)	721 (43.1)	65 (32.2)	176 (36.6)	138 (56.1)	342 (46.1)	< 0.001
Hypertension, n (%)	1572 (94.1)	183 (90.6)	447 (92.9)	230 (93.5)	712 (96.0)	0.016
Dyslipidemia, n (%)	913 (54.6)	113 (55.9)	276 (57.4)	135 (54.9)	389 (52.4)	0.381
***Medications***						
Antihypertensive agents, n (%)	1459 (87.5)	170 (84.6)	409 (85.4)	221 (89.8)	659 (88.9)	0.104
RAS inhibitor use	1077 (64.5)	140 (69.3)	287 (59.7)	176 (71.5)	474 (63.9)	0.006
Angiotensin II receptor blocker, n (%)	950 (57.0)	120 (59.7)	257 (53.7)	160 (65.0)	413 (55.7)	0.021
Angiotensin converting enzyme inhibitor, n (%)	173 (10.4)	23 (11.4)	40 (8.4)	26 (10.6)	84 (11.3)	0.376
Hypoglycemic agent, n (%)	526 (31.6)	47 (23.4)	121 (25.3)	113 (45.9)	245 (33.1)	< 0.001
Iron supplementation, n (%)	236 (14.2)	39 (19.4)	81 (16.9)	24 (9.8)	92 (12.4)	0.004
***Laboratory findings***						
Creatinine (mg/dL)	2.60 [1.88, 3.55]	2.41 [1.83, 3.19]	2.02 [1.45, 2.85]	3.52 [2.60, 4.56]	2.76 [2.10, 3.68]	< 0.001
eGFR (ml/min/1.73 m^2^)	18.15 [13.07, 25.40]	17.50 [13.14, 24.69]	19.39 [13.12, 27.27]	15.60 [11.81, 22.63]	18.65 [13.57, 24.84]	< 0.001
Hemoglobin (g/dL)	9.80 (0.88)	9.87 (0.83)	9.73 (0.89)	9.91 (0.87)	9.79 (0.88)	0.039
MCV (fL)	93.05 (5.69)	91.66 (5.61)	93.71 (5.75)	91.65 (5.54)	93.46 (5.58)	< 0.001
Fe (μg/dL)	70.62 (26.21)	67.44 (24.84)	68.30 (26.67)	74.75 (24.61)	71.59 (26.58)	0.004
Ferritin (ng/mL)	96.10 [45.60, 177.25]	60.50 [38.05, 106.25]	70.20 [37.60, 133.00]	127.50 [65.90, 213.25]	113.00 [55.40, 202.00]	< 0.001
Transferrin saturation (%)	26.14 [20.51, 32.07]	25.09 [19.13, 29.86]	24.00 [18.90, 29.82]	27.51 [23.16, 33.36]	27.02 [21.44, 33.42]	< 0.001
HbA1c (%)	6.12 (0.92)	6.40 (1.35)	6.12 (0.88)	6.09 (0.96)	6.08 (0.80)	0.014
Urinary protein-creatinine ratio (g/gCr)	1.27 [0.39, 2.94]	1.28 [0.43, 2.97]	0.80 [0.21, 2.06]	2.62 [0.88, 4.96]	1.30 [0.46, 2.81]	< 0.001

Continuous values are expressed as the mean (standard deviation) or median [interquartile range]. Discrete data are expressed as n (%). Abbreviations: n, number; %, percentage; CKD, chronic kidney disease; DA, darbepoetin alfa; eGFR, estimated glomerular filtration rate; ERI-1B, ESA resistance index -1B; ESA, erythropoiesis-stimulating agent; iEResI, initial ESA response index; MCV, mean corpuscular volume; RAS, renin-angiotensin system; U-Prot, urinary protein excretion.

### High-priority attributes for renal function deterioration and CVD events in age and sex cross-classification

**Fig. 3:**
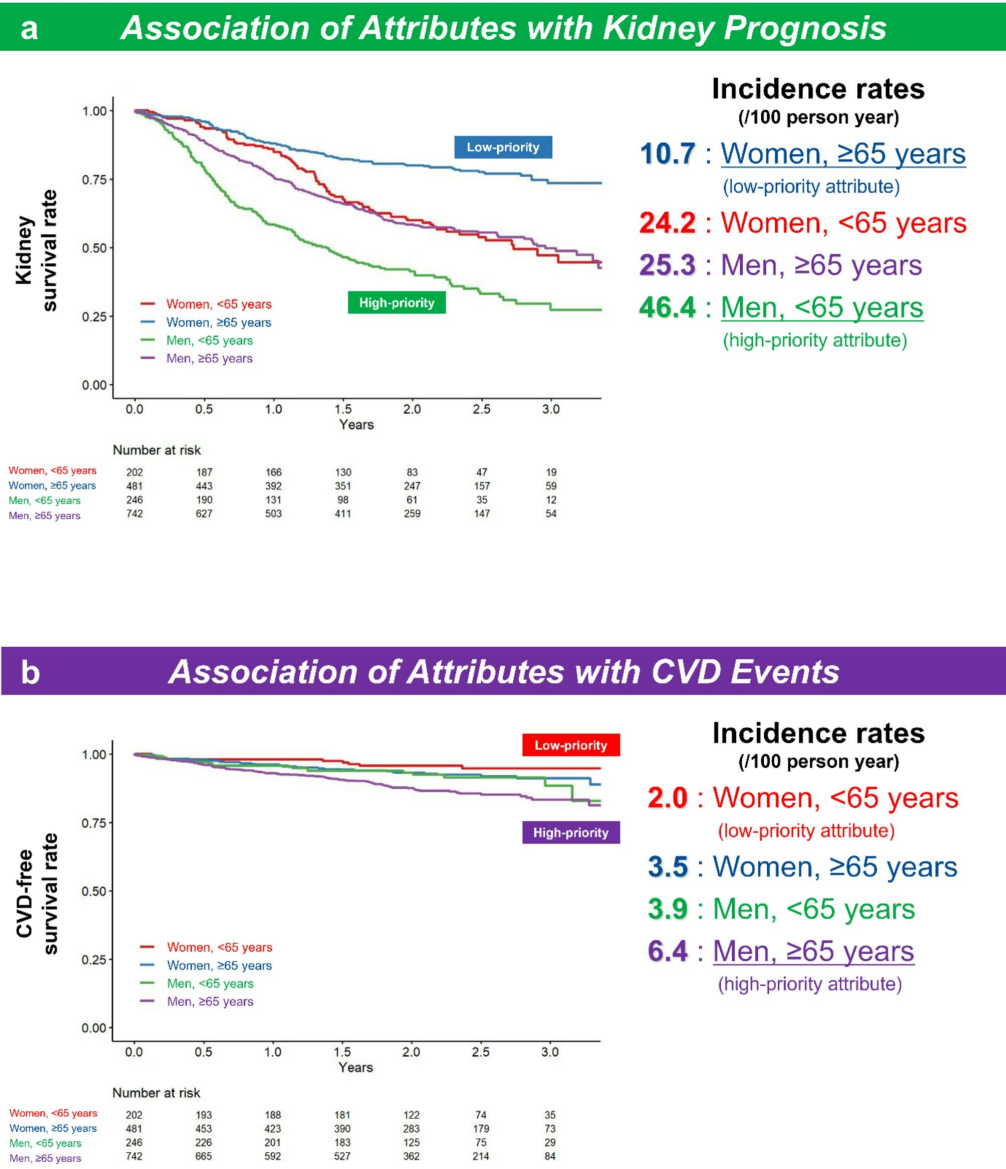
Association of attributes with kidney prognosis (**a**) and CVD events (**b**). The analysis is based on the Kaplan–Meier curves and incidence rates. **a** For kidney prognosis, male sex in those aged < 65 years was shown to be a high-priority attribute and female sex in those aged ≥ 65 years was shown to be a low-priority attribute. **b** For CVD events, male sex in those aged ≥ 65 years was shown to be a high priority attribute and male sex in those aged < 65 years was shown to be a low priority attribute. CVD, cardiovascular disease.

Kaplan–Meier curves revealed that renal outcomes were worst in men aged < 65 years and best in women aged ≥ 65 years (Fig. 3a), whereas for CVD events, prognoses were worst in men aged ≥ 65 years and best in women aged < 65 years (Fig. 3b). The incidence rate of kidney events was highest in men aged < 65 years (46.4/100 person-years) and lowest in women aged ≥ 65 years (10.7/100 person-years) compared with the other sub-cohorts (Table 2, Fig. 3a). For CVD events, the incidence rate was highest in men aged ≥ 65 years (6.4/100 person-years) and lowest in women aged < 65 years (2.0/100 person-years) (Table 3, Fig. 3b). Thus, the cross-classification approach discerned the distinction between the high-priority attributes of renal and CVD outcomes. Male sex in those aged < 65 years was identified as a high-priority attribute for renal function deterioration, whereas in those aged ≥ 65 years, it was identified as a high-priority attribute for CVD events.

**Table 2:** Incidence and Cox models for kidney events by sex and age cross-classified sub-cohorts.

Sub-cohort	Incidence rate (/100 pt-yrs) (95% CI)	Crude HR (95% CI)	*P*–value	Model 1 HR (95% CI)	*P*–value	Model 2 HR (95% CI)	*P*–value	Attribute Evaluation
Women, < 65 years (N = 202, event N = 89)	**24.2 (19.4–29.8)**	Ref		Ref		Ref		–
Women, ≥ 65 years (N = 481, event N = 102)	**10.7 (8.7–13.0)**	0.45 (0.34–0.60)	< 0.001*	0.45 (0.34–0.62)	< 0.001*	0.50 (0.37–0.68)	< 0.001*	Low-priority
Men, < 65 years (N = 246, event N = 150)	**46.4 (39.3–54.4)**	1.93 (1.48–2.51)	< 0.001*	1.82 (1.34–2.47)	< 0.001*	1.79 (1.32–2.43)	< 0.001*	High-priority
Men, ≥ 65 years (N = 742, event N = 301)	**25.3 (22.6–28.4)**	1.05 (0.83–1.33)	0.665	1.45 (1.10–1.92)	0.009*	1.41 (1.07–1.85)	0.016*	–

*P < 0.05. Model 1: Adjustment for age, sex, diabetes, hypertension, dyslipidemia, smoking, hemoglobin level at baseline, eGFR at baseline, urinary protein-to-creatinine ratio at baseline, and initial ESA response index (iEResI); Model 2: Adjustment for age, sex, diabetes, hypertension, dyslipidemia, smoking, hemoglobin level at baseline, eGFR at baseline, urinary protein-to-creatinine ratio at baseline, and ESA resistance index-1B (ERI-1B). Abbreviations: CI, confidence interval; eGFR, estimated glomerular filtration rate; ERI-1B, erythropoiesis-stimulating agent resistance index -1B; HR, hazard ratio; iEResI, erythropoiesis-stimulating agent response index.

Incidence rates are in [bold].

**Table 3:** Incidence and Cox models for cardiovascular events by sex and age sub-cohorts.

Sub-cohort	Incidence rate (/100 pt-yrs) (95% CI)	Crude HR (95% CI)	*P* value	Attribute evaluation
Women, < 65 years (N = 202, event N = 9)	**2.0 (0.9–3.8)**	ref		Low-priority
Women, ≥ 65 years (N = 481, event N = 36)	**3.5 (2.4–4.8)**	1.74 (0.84–3.60)	0.139	–
Men, < 65 years (N = 246, event N = 19)	**3.9 (2.4–6.1)**	1.94 (0.88–4.30)	0.100	–
Men, ≥ 65 years (N = 742, event N = 91)	**6.4 (5.2–7.9)**	3.16 (1.59–6.26)	0.001*	High-priority

*P < 0.05. Abbreviations: CI, confidence interval; HR, hazard ratio. Incidence rates are in [bold].

### Risk factors for renal function deterioration and CVD events

**Fig. 4:**
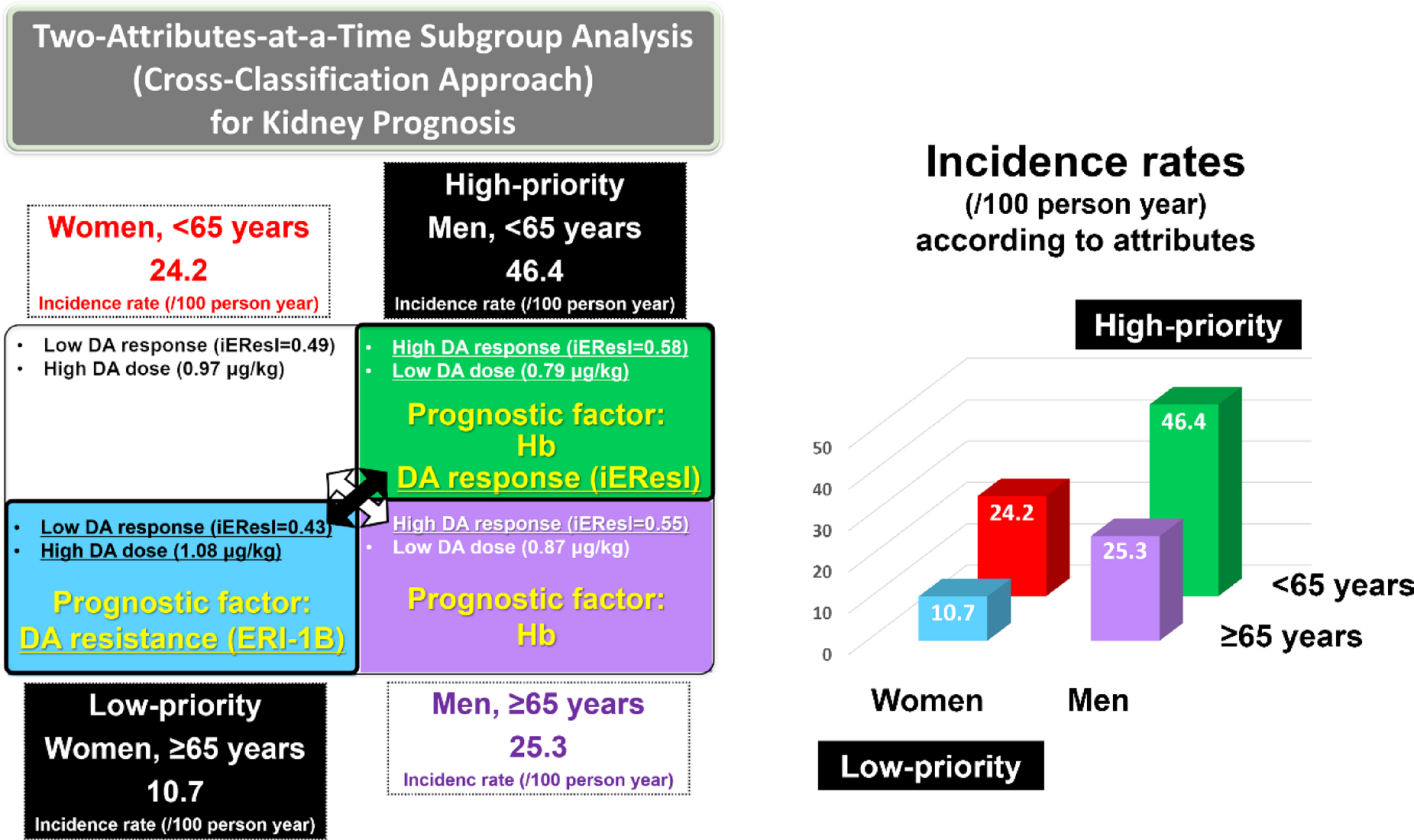
Summary of kidney prognosis based on the attribute-based cross-classification approach (Importance Grid Analysis). Kidney prognostic factors for men aged < 65 years, which are important attributes regarding kidney prognosis, were baseline Hb and DA responsiveness (the iEResI). In these attributes, DA dosage was low and DA responsiveness was high; thus, it was expected that increasing DA responsiveness through treatment, such as increasing the amount of DA or iron, would improve kidney prognosis. In contrast, a renal prognostic factor for women aged ≥ 65 years, which is a less important attribute, was DA resistance (ERI-1B). In these attributes, DA dosage was high and DA responsiveness was low; thus, it would be important to search for the cause of ESA resistance rather than strengthening treatment, such as increasing the amount of DA or iron. DA, darbepoetin alfa; Hb, hemoglobin; iEResI, initial erythropoiesis-stimulating agent response index; ERI-1B, erythropoiesis-stimulating agent resistance index-1B.

The results of the multivariable Cox proportional hazards model analyses for renal function deterioration and CVD events are presented in Table 4. Regarding kidney prognosis, multivariable Cox analyses revealed that high Hb levels (hazard ratio [HR], 0.75; *P* < 0.001) and high iEResI (HR, 0.76; *P* < 0.001) were associated with better kidney prognosis in the entire cohort (Table 4, Model A). When cross-classified, for men aged < 65 years (high-priority attribute for poor kidney outcome), high Hb levels (HR, 0.64; *P* < 0.001) and high iEResI (HR, 0.63; *P* = 0.019) were associated with better kidney prognosis (Table 5). In contrast, ERI-1B was not associated with kidney prognosis in the entire cohort (Table 4, Model B) but was associated with poor kidney prognosis (HR, 1.10; *P* < 0.001) in women aged ≥ 65 years (low priority attribute for good kidney outcome) (Table 6). High Hb levels were also associated with good kidney outcomes in men aged ≥ 65 years. The results are summarized in Fig. 4.

**Table 4:** Multivariable Cox models for CKD progression and CVD events in relation to risk factors (N = 1671).

	Model A-iEResI: Related factors for CKD (with iEResI)		Model B- ERI-1B: Related factors for CKD (with ERI-1B)		Model C-iEResI: Related factors for CVD (with iEResI)		Model D- ERI-1B: Related factors for CVD (with ERI-1B)	
Variable	Hazard ratio (95% CI)	*P*-value	Hazard ratio (95% CI)	*P*-value	Hazard ratio (95% CI)	*P*-value	Hazard ratio (95% CI)	*P*-value
Age (1-year increments)	0.98 (0.98–0.99)	< 0.001*	0.99 (0.98–0.99)	< 0.001*	1.04 (1.02–1.07)	< 0.001*	1.04 (1.03–1.06)	< 0.001*
Men (vs. women)	2.47 (1.98–3.07)	< 0.001*	2.26 (1.82–2.81)	< 0.001*	1.63 (1.00–2.63)	0.048*	1.45 (0.92–2.30)	0.107
Diabetes, no	1.31 (1.08–1.57)	0.005*	1.27 (1.06–1.53)	0.010*	0.74 (0.51–1.07)	0.114	0.67 (0.47–0.96)	0.027*
Hypertension, no	0.70 (0.41–1.19)	0.189	0.80 (0.51–1.26)	0.335	1.25 (0.45–3.46)	0.669	1.46 (0.67–3.22)	0.343
Dyslipidemia, no	1.00 (0.84–1.19)	0.980	0.99 (0.83–1.18)	0.910	1.05 (0.73–1.52)	0.780	1.25 (0.88–1.77)	0.212
Smoking status (current or ever), no	0.96 (0.79–1.17)	0.703	1.00 (0.82–1.22)	0.982	1.27 (0.82–1.97)	0.287	1.37 (0.90–2.08)	0.144
eGFR at baseline (1 mL/min/1.73 m^2^ increase)	0.88 (0.86–0.89)	< 0.001*	0.88 (0.87–0.89)	< 0.001*	0.97 (0.95–0.99)	0.012*	0.97 (0.95–0.99)	0.006*
Log (Urinary protein–creatinine ratio at baseline)	1.17 (1.15–1.20)	< 0.001*	1.18 (1.15–1.21)	< 0.001*	1.07 (1.00–1.13)	0.073	1.07 (1.01–1.13)	0.022*
Hemoglobin at baseline (1 g/dL increase)	0.75 (0.64–0.89)	< 0.001*	0.82 (0.71–0.90)	0.009*	0.83 (0.68–1.02)	0.073	0.90 ((0.74–1.10)	0.289
iEResI	0.76 (0.64–0.89)	< 0.001*	NA	NA	0.97 (0.76–1.24)	0.816	NA	NA
ERI-1B	NA	NA	0.97 (0.93–1.01)	0.087	NA	NA	1.03 (0.99–1.08)	0.172

*P < 0.05. Variables of candidate risk factors of CKD and CVD, ERI-1B, and the iEResI were included in the multivariable models. Abbreviations: CI, confidence interval; CKD, chronic kidney disease; CVD, cardiovascular disease; eGFR, estimated glomerular filtration rate; ERI-1B, erythropoiesis-stimulating agent resistance index -1B; iEResI, erythropoiesis-stimulating agent response index; n, number; NA, not applicable; *P*, calculated probability.

**Table 5:** Multivariable Cox models for CKD progression and risk factors (iEResI by sex and age sub-cohorts).

*P*-Value for interaction = 0.003* (iEResI × sex and age category)	Model A-iEResI: Related factors for CKD (Men, < 65 years) N = 246, event N = 150		Model A-iEResI: Related factors for CKD (Women, < 65 years) N = 202, event N = 89		Model A-iEResI: Related factors for CKD (Men, ≥ 65 years) N = 742, event N = 301		Model A-iEResI: Related factors for CKD (Women, ≥ 65 years) N = 481, event N = 102	
Variable	Hazard ratio (95% CI)	*P*-value	Hazard ratio (95% CI)	*P*-value	Hazard ratio (95% CI)	*P*-value	Hazard ratio (95% CI)	*P*-value
Age (1-year increments)	1.00 (0.98–1.02)	0.990	1.00 (0.97–1.03)	0.974	1.01 (0.99–1.03)	0.378	0.98 (0.94–1.01)	0.139
Diabetes, no	1.41 (0.94–2.10)	0.094	1.15 (0.62–2.14)	0.652	1.33 (1.02–1.73)	0.033*	1.17 (0.72–1.89)	0.527
Hypertension, no	1.38 (0.57–3.33)	0.471	0.29 (0.08–1.02)	0.055	1.02 (0.25–4.15)	0.980	0.37 (0.11–1.25)	0.110
Dyslipidemia, no	0.85 (0.58–1.27)	0.434	1.74 (1.03–2.95)	0.038*	0.89 (0.69–1.15)	0.380	1.22 (0.75–1.92)	0.436
Smoking status (current or ever), no	0.78 (0.51–1.19)	0.240	1.37 (0.79–2.36)	0.265	0.95 (0.72–1.26)	0.734	0.61 (0.30–1.24)	0.170
eGFR at baseline (1 mL/min/1.73 m^2^ increase)	0.91 (0.88–0.94)	< 0.001*	0.87 (0.83–0.91)	< 0.001*	0.86 (0.84–0.88)	< 0.001*	0.86 (0.82–0.89)	< 0.001*
Log (Urinary protein–creatinine ratio at baseline)	1.12 (1.06–1.18)	< 0.001*	1.16 (1.09–1.25)	< 0.001*	1.26 (1.21–1.31)	< 0.001*	1.22 (1.13–1.30)	< 0.001*
Hemoglobin at baseline (1 g/dL increase)	0.64 (0.51–0.80)	< 0.001*	0.90 (0.66–1.25)	0.535	0.81 (0.70–0.93)	0.003*	0.74 (0.57–0.96)	0.021*
iEResI	0.63 (0.43–0.93)	0.019*	0.77 (0.40–1.89)	0.432	0.87 (0.71–1.07)	0.194	0.63 (0.40–1.00)	0.050

*P < 0.05. Variables of candidate risk factors of CKD and the iEResI were included in the multivariable models. Abbreviations: CI, confidence interval; CKD, chronic kidney disease; eGFR, estimated glomerular filtration rate; iEResI, erythropoiesis-stimulating agent response index; n, number; *P*, calculated probability.

**Table 6:** Multivariable Cox models for CKD progression and risk factors (ERI-1B by sex and age sub-cohorts).

*P*-Value for interaction = 0.020* (ERI-1B × sex and age category)	Model B- ERI-1B: Related factors for CKD (Men, < 65 years) N = 246, event N = 150		Model B- ERI-1B: Related factors for CKD (Women, < 65 years) N = 202, event N = 89		Model B- ERI-1B: Related factors for CKD (Men, ≥ 65 years) N = 742, event N = 301		Model B- ERI-1B: Related factors for CKD (Women, ≥ 65 years) N = 481, event N = 102	
Variable	Hazard ratio (95% CI)	*P*-value	Hazard ratio (95% CI)	*P*-value	Hazard ratio (95% CI)	*P*-value	Hazard ratio (95% CI)	*P*-value
Age (1-year increments)	1.00 (0.98–1.02)	0.668	0.99 (0.95–1.02)	0.401	1.01 (0.99–1.03)	0.384	0.99 (0.95–1.02)	0.401
Diabetes, no	1.44 (0.97–2.14)	0.071	1.00 (0.52–1.92)	0.995	1.22 (0.94–1.57)	0.127	1.09 (0.68–1.75)	0.710
Hypertension, no	1.76 (0.83–3.75)	0.143	0.24 (0.07–0.82)	0.023*	1.15 (0.47–2.85)	0.756	0.64 (0.22–1.86)	0.415
Dyslipidemia, no	0.84 (0.57–1.22)	0.357	1.48 (0.87–2.52)	0.148	0.86 (0.66–1.11)	0.242	1.45 (0.93–2.25)	0.098
Smoking status (current or ever), no	0.81 (0.53–1.23)	0.319	1.28 (0.75–2.19)	0.374	1.05 (0.80–1.39)	0.714	1.15 (0.63–2.10)	0.642
eGFR at baseline (1 mL/min/1.73 m^2^ increase)	0.91 (0.88–0.94)	< 0.001*	0.86 (0.82–0.90)	< 0.001*	0.86 (0.84–0.88)	< 0.001*	0.86 (0.83–0.90)	< 0.001*
Log (Urinary protein–creatinine ratio at baseline)	1.12 (1.07–1.18)	< 0.001*	1.15 (1.06–1.25)	< 0.001*	1.26 (1.21–1.31)	< 0.001*	1.22 (1.14–1.30)	< 0.001*
Hemoglobin at baseline (1 g/dL increase)	0.73 (0.59–0.91)	0.005*	0.80 (0.59–1.09)	0.155	0.82 (0.71–0.95)	0.009*	0.93 (0.70–1.24)	0.628
ERI-1B	1.02 (0.96–1.08)	0.612	0.98 (0.90–1.07)	0.691	0.97 (0.93–1.01)	0.087	1.10 (1.04–1.16)	< 0.001*

*P < 0.05. Variables of candidate risk factors of CKD and ERI-1B were included in the multivariable models. Abbreviations: CI, confidence interval; CKD, chronic kidney disease; eGFR, estimated glomerular filtration rate; ERI-1B, erythropoiesis-stimulating agent resistance index -1B; n, number; *P*, calculated probability.

Regarding CVD events, multivariable Cox analyses revealed that Hb levels, iEResI, and ERI-1B were not associated with CVD events in the entire cohort (Table 4, Models C and D). As the incidence of CVD events was low, multivariable Cox analysis based on cross-classification was not performed.

### Significance of ERI-1B ≥ 5.2 in the cross-classification by age and sex

**Fig. 5:**
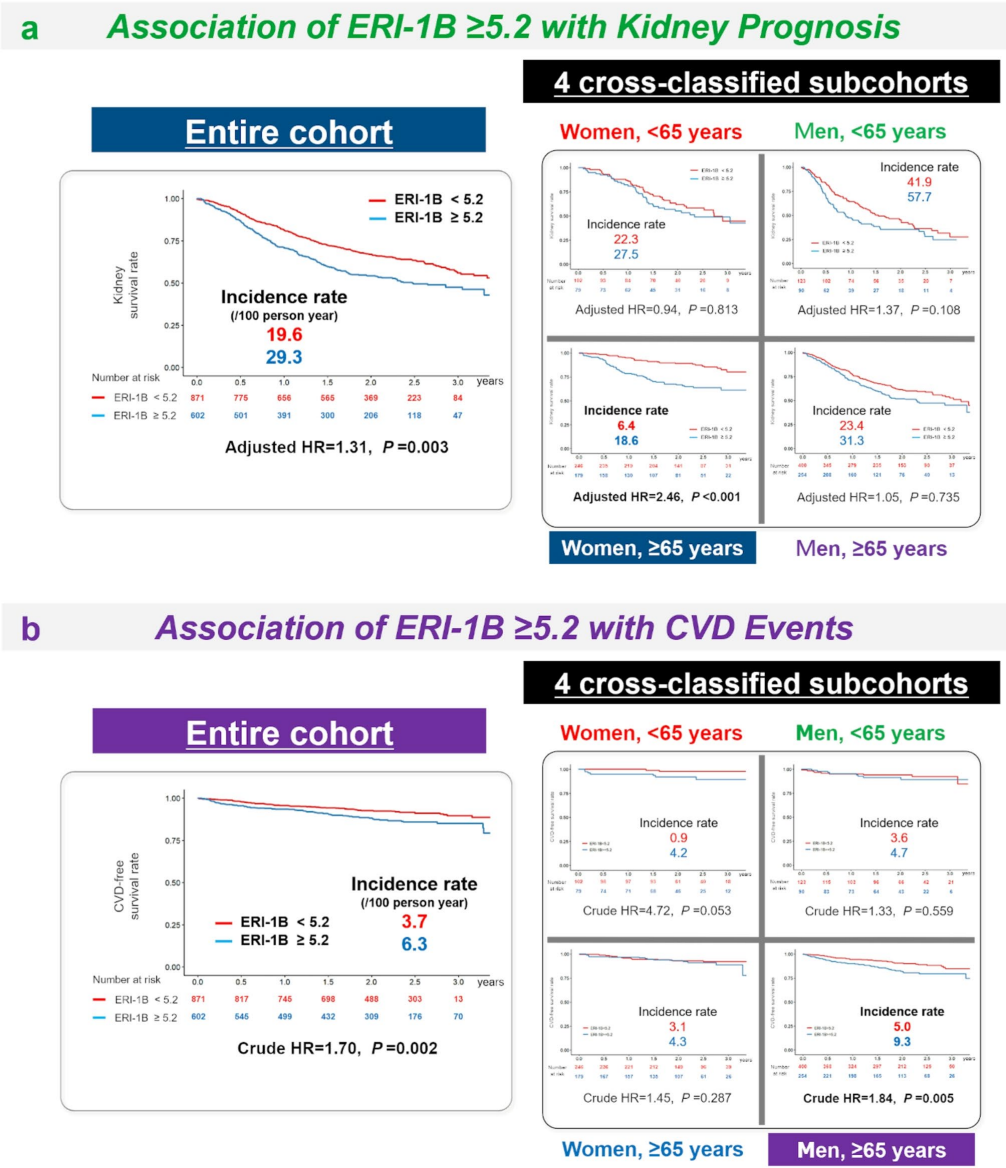
Association of an ERI-1B ≥ 5.2 with kidney prognosis (**a**) and CVD events (**b**). The analysis is based on Kaplan–Meier curves and incidence rates in the entire cohort and four age-and-sex cross-classified sub-cohorts. **a** The hazard ratio was adjusted for age, sex, diabetes, hypertension, dyslipidemia, smoking, hemoglobin level at baseline, eGFR, and urinary protein-to-creatinine ratio. For kidney prognosis, an ERI-1B ≥ 5.2 was a significant related factor in the entire cohort and a low-priority attribute in women aged ≥ 65 years. **b** The hazard ratio was not adjusted. For CVD events, an ERI-1B ≥ 5.2 was a significant related factor in the entire cohort and a high-priority attribute in men aged ≥ 65 years. ERI-1B, erythropoiesis-stimulating agent resistance index-1B; HR, hazard ratio; CVD, cardiovascular disease; eGFR, estimated glomerular filtration rate.

Finally, we conducted a subgroup analysis using cross-classification based on age and sex to evaluate the effectiveness of the ERI-1B cutoff values calculated in the preceding study^36^ (Fig. 5). Consistent with previous findings, an ERI-1B ≥ 5.2 was significantly associated with poor kidney prognosis and CVD events in the entire cohort. Within the age and sex cross-classification, an ERI-1B ≥ 5.2 was significantly associated with poor kidney prognosis, especially in women aged ≥ 65 years (Fig. 5a), and with CVD events, especially in men aged ≥ 65 years (Fig. 5b).

## Discussion

This attribute-based study, using cross-classification by sex and age, identified distinct attributes linked to poor renal and cardiovascular prognoses, with young men being associated with poor kidney prognoses and older men with poor cardiovascular prognoses (as shown in Figs. 3, 4 and Table 2–3). It also revealed that factors related to kidney prognosis differ between young men (ESA response: the iEResI) and older women (ESA resistance: ERI-1B), highlighting the importance of considering specific attributes in treating anemia in patients with CKD (as shown in Table 5 and Table 6). Thus, ABM, using cross-classification by sex and age, may be effective for treating renal anemia. Furthermore, interestingly, regarding anemia treatment-related factors, the iEResI, which is modifiable by anemia treatment, was associated with kidney outcomes in the entire cohort, whereas ERI-1B was not (as shown in Table 4, Models A and B).

Anemia increases the risk of disease progression^39^ and CVD events^40^ in patients with CKD. However, the optimal Hb target for anemia treatment in patients with CKD remains controversial^41^, and the appropriate Hb level at which to initiate treatment has not yet been fully established. Large clinical trials have demonstrated an increased risk of adverse events associated with Hb normalization^42,43^. However, secondary analyses suggest that these poor outcomes may be attributed to patient-related factors contributing to ESA hyporesponsiveness, high-dose ESA toxicities, or a combination of both rather than to elevated Hb levels^34,44,45^. Thus, Hb target-based strategies are complicated by individual hematopoietic responses^45^.

The BRIGHTEN study established the ESA resistance and response indices^34^. ERI-1B is an indicator of ESA resistance and is reportedly associated with age, comorbidities, inflammation, and malnutrition^34,46^. The iEResI is an indicator of the ESA response and is associated with low Hb levels, male sex, iron supplementation, and DA administration frequency, whereas high C-reactive protein levels, low eGFR, and high urinary Protein:Creatinine Ratio are negatively associated^34^. Thus, the iEResI is a potential indicator of “anemia treatment adequacy.” High eGFR and/or high Hb levels (reflecting good kidney function or absence of anemia) were associated with a low iEResI (indicating low ESA responsiveness or a lower need for ESA or iron). In contrast, low eGFR and/or low Hb levels (reflecting poor kidney function or presence of anemia) were associated with a high iEResI, suggesting that iEResI may serve as an indicator for identifying patients who require ESAs and/or iron supplementation. Furthermore, iron and ESA supplementation were associated with an increased iEResI^34^, and the present study demonstrated that an increased iEResI was associated with a good kidney prognosis (as shown in Table 4, Model A). In contrast, considering that a high iEResI was associated with low C-reactive protein levels^34^, a decreased iEResI is associated with ESA resistance. Thus, the iEResI value has two elements: ESA response and ESA resistance. This ambiguity makes it difficult to establish a clear cutoff value for iEResI for kidney prognosis or CVD events, unlike ERI-1B^36^.

A distinctive feature of the current study is the use of attribute-based medical research through cross-classification analysis. Cross-classification analysis revealed that iEResI was significantly associated with renal outcomes in young men (high-priority attribute for poor kidney outcome) with the poorest prognosis (as shown in Table 5), whereas ERI-1B was significantly associated with renal outcomes in older women (low-priority attribute for good kidney outcome) with the best prognosis (as shown in Table 6). These findings suggest that ESA responsiveness is a more critical factor than ESA resistance in kidney prognosis in patients with CKD and anemia. Conversely, the Kaplan–Meier survival curve indicated that older female patients with an ERI-1B ≥ 5.2 had a significantly poorer kidney prognosis (as shown in Fig. 5a), highlighting the need to identify the cause of ESA resistance in this group.

Further detailed consideration of poor kidney prognosis in young male patients with CKD and renal anemia is needed. The current study, following Japanese guidelines and including data from 168 facilities, highlighted that young male patients who were initiated on ESA with low Hb levels and low eGFR with low dose of ESA (mean age, 52.2 years old; mean Hb, 9.9 g/dL; eGFR, 15.6 mL/min/1.73 m^2^; DA during 12 weeks, 0.79 μg/kg) had worse renal outcomes (incidence rate, 46.4/100 person-years) (as shown in Table 1 and Table 2). Interestingly, although younger men had the poorest renal prognosis, they also showed the highest responsiveness to ESA treatment (as shown in Table 1: iEResI = 0.58), suggesting that suboptimal treatment intensity—rather than inherent resistance—may underlie their poor outcomes. These findings suggest that younger men with good ESA responsiveness may not receive adequate treatment until their renal function significantly deteriorates, potentially because of adherence to sex-neutral anemia treatment guidelines. Guidelines have recommended initiating treatment when the Hb level is < 10 or 11 g/dL, aiming to maintain it at ≤ 12 or 13 g/dL^31,47–52^; nevertheless, it remains unclear whether these criteria are suitable for younger men. The role of chronic hypoxia in the tubulointerstitium as the final common pathway leading to end-stage renal failure has been highlighted^53^. In young men with larger body size and a higher risk of lifestyle-related diseases, the metabolic demands on renal tubular cells are increased, which may lead to relative renal hypoxia. Clinical research on ABM can shed light on this issue.

Unlike traditional one-variable subgrouping, our cross-classification approach uncovered clinically meaningful interactions—such as the combination of high ESA responsiveness and poor renal prognosis in younger men—that may be overlooked by current guidelines using uniform thresholds. This highlights the added value of ABM in identifying patient attributes that may benefit from tailored therapeutic strategies.

Our findings highlight the need for specific Hb criteria for initiating and targeting anemia treatment in young men. Future strategies may include earlier treatment initiation^54,55^ or more tailored approaches^41,56,57^ based on patient attributes—such as raising Hb thresholds or target Hb levels specifically for younger men, and incorporating iron dynamics and inflammatory markers into ABM-based strategies^34^. To confirm these insights, interventional studies stratified by cross-classified patient characteristics (e.g., sex and age) are needed to validate ABM-based anemia management strategies.

In addition, our study revealed sex and age differences in the relationship between anemia and CKD/CVD (as shown in Figs. 3, 4 and Table 2–3), which may reflect underlying mechanisms of the cardiorenal syndrome. Recently, there has been growing interest in this syndrome^58–63^, where dysfunction in one organ system contributes to dysfunction in the other. While acknowledging the importance of these interactions, we emphasize the need for individualized treatment strategies tailored to patient characteristics—such as age and sex—to improve outcomes in both CKD and CVD.

Despite these notable strengths, the present study had several limitations. First, it was designed as an observational cohort study and involved a post-hoc analysis of the BRIGHTEN study data. Second, the administration of ESAs and iron supplementation was left to the attending physician’s discretion, which may have introduced bias. Third, although factors such as age and sex could influence the relationship between iron metabolism and CKD or CVD, this study did not evaluate iron metabolism parameters, in line with previously published findings^34^. Fourth, residual confounding factors related to renal outcomes and mortality may have remained unaccounted for in the present study. Fifth, because few medical studies have used cross-classification, evidence on the clinical characteristics and treatment needs of young men remains limited and unclear. These findings are exploratory and based on post-hoc analyses, with a potential risk of multiple testing bias. Therefore, validation through prospective studies is needed before any changes to treatment thresholds can be justified. Finally, all the participants were Japanese, which may limit the generalizability of our findings to other populations.

## Conclusions

In summary, by employing cross-classification based on sex and age, this study identified distinct attributes linked to poor renal and cardiovascular prognoses in patients with CKD. Poor kidney prognosis was more likely in younger men, while poor cardiovascular prognosis was more likely in older men. This study highlighted the importance of considering specific attributes in treating anemia, considering that kidney prognosis factors differ between young men (ESA response index: the iEResI) and older women (ESA resistance index: ERI-1B). Anemia treatment may improve kidney prognosis, especially in men aged < 65 years (a high-priority attribute of poor kidney outcome). The findings from the BRIGHTEN study are expected to significantly affect future nephrology practice.

## Data Availability

The data that support the findings of this study are available from the data center for this study, the Translational Research Center for Medical Innovation, Kobe, Japan (https://www.tri-kobe.org/), or from the supporting information files from a previous study^36^, but restrictions apply to the availability of these data, which were used under license for the current study, and so are not publicly available. Data are however available from the corresponding author upon reasonable request and with permission of the Translational Research Center for Medical Innovation.

## Abbreviations

ABM: Attribute-based medicine
CKD: Chronic kidney disease
CVD: Cardiovascular disease
DA: Darbepoetin alfa
eGFR: Estimated glomerular filtration rate
ERI-1B: ESA resistance index-1B
ESA: Erythropoiesis-stimulating agent
Hb: Hemoglobin
iEResI: Initial ESA response index
UMIN-CTR: University Hospital Medical Information Network Clinical Trials Registry
